# Expected Valence Predicts Choice in a Recurrent Decision Task

**DOI:** 10.3389/fnins.2020.580970

**Published:** 2020-11-26

**Authors:** Daniel T. Jäger, Melanie Boltzmann, Jens D. Rollnik, Jascha Rüsseler

**Affiliations:** ^1^Department of Psychology, Otto-Friedrich University of Bamberg, Bamberg, Germany; ^2^Bamberg Graduate School of Affective and Cognitive Science (BaGrACS), Otto-Friedrich University of Bamberg, Bamberg, Germany; ^3^Institute for Neurorehabilitative Research, BDH-Clinic Hessisch Oldendorf, Hessisch Oldendorf, Germany

**Keywords:** affect, decision, predecisional, expected valence, anticipation, goal-directed emotion, fMRI, Iowa Gambling Task

## Abstract

There is empirical evidence that expected yet not current affect predicts decisions. However, common research designs in affective decision-making show consistent methodological problems (e.g., conceptualization of different emotion concepts; measuring only emotional valence, but not arousal). We developed a gambling task that systematically varied learning experience, average feedback balance and feedback consistency. In Experiment 1 we studied whether predecisional current affect or expected affect predict recurrent gambling responses. Furthermore, we exploratively examined how affective information is represented on a neuronal level in Experiment 2. Expected and current valence and arousal ratings as well as Blood Oxygen Level Dependent (BOLD) responses were analyzed using a within-subject design. We used a generalized mixed effect model to predict gambling responses with the different affect variables. Results suggest a guiding function of expected valence for decisions. In the anticipation period, we found activity in brain areas previously associated with valence-general processing (e.g., anterior cingulate cortex, nucleus accumbens, thalamus) mostly independent of contextual factors. These findings are discussed in the context of the idea of a valence-general affective work-space, a goal-directed account of emotions, and the hypothesis that current affect might be used to form expectations of future outcomes. In conclusion, expected valence seems to be the best predictor of recurrent decisions in gambling tasks.

## Introduction

According to Prospect Theory (Kahneman and Tversky, 2013), human decision-making is not solely rational but rather subject to inherent biases that influence judgement, decision-making, and human behavior. Other authors have also suggested an affective involvement in decision-making and behavior regulation (Mellers et al., 1997; Loewenstein and Lerner, 2003; Lerner et al., 2015; DeWall et al., 2016). However, the exact role of emotions in decision-making and behavior regulation is the subject of ongoing debate. One important issue in this context is how to conceptualize different components of emotions. In our opinion, there are two dimensions that need to be separated. First, it appears useful to differentiate between pre- and post-decisional emotions (Mellers et al., 1997). *Predecisional emotions* are present before the decision is made while *postdecisional emotions* arise after the decision when experiencing the feedback. Second, Loewenstein and Lerner (2003) broadly distinguished expected from immediate emotions. *Expected emotions* refer to the prediction of future emotional consequences depending on the respective decision or action while *current/immediate emotions* refer to emotions that are present while the decision is made.

In our view, distinguishing between expected and current emotions also benefits research concerning the role of predecisional emotions in recurrent decision-making. This distinction mirrors dual process accounts that have been proposed in the decision-making literature (Lerner et al., 2015; Beer, 2017). These accounts propose that decision-making consists of two kinds of processes. First, cognitive processes that require time, deliberation, and cognitive resources; and, second, automatic processes that work in a quick and dirty fashion and thereby incorporate current emotions as a mediating variable between stimulus and response. In a meta-analysis, DeWall et al. (2016) examined whether during the anticipation period current emotions or expected emotions (they called it anticipated emotions) guide decisions and behavior. They concluded that there is weak evidence to support the claim that current emotions cause decisions but stronger preliminary evidence that expected emotions do so. This contradicts the default assumption of the described dual process accounts which assume that current emotions directly cause behavior (e.g., Loewenstein et al., 2001). However, DeWall et al. (2016) did not pit each theory against each other but rather tested them separately. Furthermore, they included studies in their analysis that asked about distinct emotion categories and, therefore, for conscious emotions. We think that this level of analysis might neglect causal mechanisms among emotion components. Thus, we propose to look at emotion components and causal mechanisms among them. For example, it could be fruitful to examine subjective feelings and how they relate to decisions as, for example, Charpentier et al. (2016) did. They showed that feelings could predict choices in a gambling task better than a value-based prediction model. However, they did not use a two dimensional feelings model but just measured expected valence. Barrett and Bliss-Moreau (2009) argued that the core of generating subjective feelings relies on two affect dimensions: *valence or pleasantness* and *arousal or activation* (Feldman and Russell, 1998). Thus, they suppose that humans continuously monitor how pleasant and arousing something is and use this to construct an emotional episode. In sum, Charpentier et al. (2016) have not taken a two-dimensional perspective on feelings as they neglected arousal in their experiments. Moreover, they did not investigate? how predecisional current affect and expected affect relate to one another and the respective decision.

Decision Affect Theory offers a theoretical foundation for the role of anticipated pleasure in choice prediction (Mellers et al., 1999; Mellers and McGraw, 2001). Simply put, this theory posits that “… when making decisions, people anticipate the pleasure or pain of future outcomes, weigh those feelings by the chances they will occur, and select the option with greater average pleasure” (Mellers and McGraw, 2001, p. 210). In several experiments they have identified several contextual factors which influence anticipated pleasure ratings. They used pie charts and, therefore, fully displayed associated probabilities and outcomes. Moreover, participants received information about their unchosen options. Each decision participants made referred to a new gambling situation with different probabilities and outcomes. Hence, participants knew probabilities in advance, could not learn from feedback, and could not avoid gambling. Based on this experimental paradigm, the authors identified four effects which influenced anticipated pleasure ratings. First, outcome effects (the higher the outcome, the higher anticipated pleasure ratings and vice versa). Second, suprise effects (the less probable an outcome the more pronounced are outcome effects). Last, regret and disappointment effects can be subsumed under comparison effects which show that the unobtained outcomes or unchosen outcomes also influence anticipated pleasure ratings. Finally, the authors could show that expected pleasure ratings were correlated with decisions participants made (Mellers and McGraw, 2001).

Another common method to investigate the role of emotions in decision-making is the Iowa Gambling Task (IGT; Aram et al., 2019). Participants have to draw a card from one of four decks. They do not know that there are good and bad decks. Bad decks produce high wins in the short term but on average losses in the long term as possible losses are also higher. However, good decks result in small wins in the short term but on average wins in the long term as possible losses are even smaller. Participants have to figure this out via trial and error. Patients with damaged prefrontal brain regions performed worse in the IGT and did not show increased anticipatory autonomous activity before making their decision. In contrast, neurologically healthy adults displayed an increase in electrodermal activity prior to a decision that was present even before participants gained conscious insight into the task structure (Bechara et al., 1997). Consequently, results suggest that electrophysiological correlates, which could be termed as current affect or somatic markers, as Bechara and Damasio (2005) call it, are essential to advantageous decision-making. At the same time, the interpretation of results and the task design have been criticized and alternative explanations have been proposed (Dunn et al., 2006). We want to highlight two major points regarding IGT’s task design: decks are not presented in a counterbalanced order and all four decks are presented simultaneously which makes it impossible to see which deck is attended. If feelings guide choices, knowing which deck participants focus attention on, is crucial as several expected and current feelings might be present at the same time.

Taken together, previous research in choice prediction has neglected the two-dimensional nature of affect (Mellers and McGraw, 2001; Charpentier et al., 2016) or used only clustered data for choice prediction (Schlösser et al., 2013). As described, research has produced inconsistent findings. Additionally, some experiments conducted in this area of research used gambling tasks with fully displayed probabilities for each choice option and did not incorporate learning experience. In more ecologically valid tasks like the IGT, choice prediction based on predecisional subjective feelings has to our knowledge not been employed. As we wanted to understand causal mechanisms among emotional components on a subjective and neuronal level of analysis, we measured both dimensions of affect (arousal and valence) and examined both expected and current affect. To get an understanding of how contextual factors translate into emotion components and neural activations in a recurrent decision task, we designed two experiments. In Experiment 1 we examined how the proposed constructs are influenced by contextual factors and which feeling constructs predict choice best. In Experiment 2 we tried to replicate Experiment 1’s main findings regarding contextual influences on predecisional affective constructs. At the same time, we exploratively looked at brain activity of our gambling task to get a preliminary understanding of how contextual factors might influence brain activity and predecisional affect ratings.

## Experiment 1

We designed a gambling task that was similar to the IGT in the way that participants had to make recurrent decisions and did not know gambling probabilities in advance. We did so to address the previously mentioned shortcomings of the IGT. At the same time our task had a similar ecologically valid structure as the IGT as outcome probabilities were unknown in advance (like most times in real life), participants could avoid certain outcomes, and they had to adapt their behavior based on previous experiences.

Thus, our task presented only one choice option at a time and allowed to vary contextual factors like feedback consistency, learning experience (time), and feedback balance in a systematic way. Feedback consistency refers to the probability of certain outcomes (see Table 1), feedback balance refers to the average outcome that could be obtained (see Table 1), and learning experience refers to three different time points we took measurements of predecisional affect. Four different symbols were used in the task. Each symbol had a unique pay-off schedule that was unknown to the participants. For each symbol, participants could decide whether they wanted to gamble or not. If participants decided not to gamble, their current balance remained unaffected. Thus, participants had the option of avoiding certain actions. If participants decided to gamble, however, they could win or lose points. Two symbols returned consistent positive or negative feedback while the other two symbols returned inconsistent feedback. Furthermore, the overall balance was positive for two symbols and negative for the other two symbols.

**TABLE 1:** Example of Symbol-Feedback contingencies depending on the average feedback balance and the feedback consistency in Experiment 1.

Average feedback balance	Feedback consistency	Symbol	P_(+15 points)_ (%)	P_(–15 points)_ (%)
Positive	Consistent	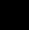	100	0
	Inconsistent	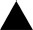	66.6	33.3
Negative	Consistent	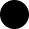	0	100
	Inconsistent	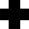	33.3	66.6

*P_(+15 points)_ refers to the probability of winning 15 points and P_(–15 points)_ to the complementary probability of losing 15 points if the participant decided to gamble. Please note that symbol-feedback condition mapping was randomly assigned for each participant.*

To better understand the affective involvement in our task and how affective components develop with task experience, we measured different kinds of affect at three different time-points. We took a two-dimensional perspective and, therefore, measured current valence and current arousal. Additionally, we looked at the expected valence and expected arousal for each option (gambling, passing). In a first step we analyzed whether the proposed affect constructs were sufficiently different from one another. Second, we wanted to show that contextual factors like feedback balance, feedback consistency, and learning experience had an effect on self-reported affect constructs. Finally, we hypothesized in line with the previously presented research (Mellers and McGraw, 2001; Dunn et al., 2006; Charpentier et al., 2016; DeWall et al., 2016) that expected affect and especially expected valence are better predictors for decision-making than current affect.

### Materials and Methods

#### Participants

Data were collected from 25 healthy adults (*M*_age_ = 24.1 years, *SD* = 3.6 years, 10 men). Participants had normal or corrected to normal vision; 21 participants were right-handed, four were left-handed; all participants were students at the University of Bamberg and received course credit for participation. The study was conducted in accordance with the Declaration of Helsinki. Participants gave their written informed consent and were told that they could refrain from the study at any point without consequences. The study protocol was approved by the local ethics commission.

#### Materials

The experiment consisted of two types of blocks: Learning Blocks and Predecisional Affective Questionnaire (PAQ) Blocks. For stimulus presentation, we used the software NBS Presentation^1^. For answer collection, we used a two keyed Cedrus Response Box (RB-380) and paper-pencil questionnaires.

##### Gambling task in the learning blocks

**FIGURE 1:**
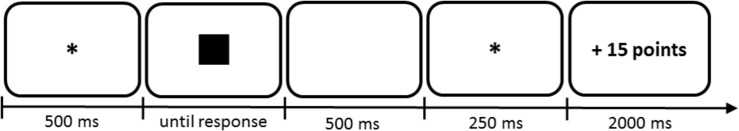
Example trial to illustrate the timing of the Gambling Task. Numbers characterize presentation durations in ms. In this case the participant would have chosen to gamble and subsequently won 15 points. ITI = 500 ms. ^∗^ Indicates a fixation dot.

**FIGURE 2:**
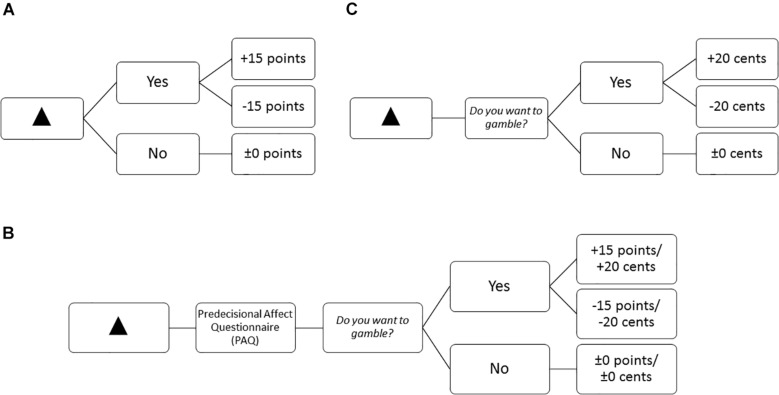
Example of the trial structure and possible feedback depending on gambling decision for a positive-inconsistent symbol **(A)** in the learning blocks in Experiment 1, and **(B)** the questionnaire blocks for both experiments (Participants could win or lose points in Experiment 1 and cents in Experiment 2), and **(C)** the learning and fMRI blocks in Experiment 2.

The main goal of the gambling task for participants was to maximize points. Each participant started with a balance of 500 points. By making advantageous decisions participants could accumulate wins and avoid losses. In each trial of the gambling task, one of four symbols (circle, triangle, square, cross) was displayed (see Table 1). The presented symbol served as a clue for the possible feedback based on previous experience the participants made with this symbol (see Figure 1 for timing parameters, ITI = 500 ms, and Figure 2A for trial structure). Thus, in each trial the participant had to decide whether she wanted to gamble or have a pass on the symbol. If the participant decided not to gamble (pass), the feedback was always ± 0 points irrespectively of the previously presented symbol. If the participant decided to gamble, feedback was determined based on constant symbol dependent winning and losing probabilities (see Table 1 for an example). Moreover, participants were not told a symbol’s objective winning probability. They were rather instructed to figure out via trial and error for which symbol they expected a positive point balance. Symbol–probability pairings depended on two factors (average feedback balance, average feedback consistency) and were randomly assigned for each participant (see Table 1 for an example and Table 2 for an overview). The factor feedback balance coded whether symbols yield positive or negative feedback on average. The factor feedback consistency coded whether symbols returned consistent or inconsistent/mixed feedback. Hence, there were three possible outcomes depending on the previously presented symbol and the decision. In our example from Table 1: the square had a 100% probability of winning 15 points (and 0% losing probability); the circle had a 66.6% probability of winning 15 points (33.3% losing probability); the cross had a 33.3% probability of winning 15 points (66.6% losing probability); the circle had a 0% probability of winning 15 points (100% losing probability). Thus, to maximize gains, participants should gamble when experiencing an overall positive balance (square and triangle) and should avoid gambling when experiencing an overall negative balance (circle and cross). In each block the two symbols producing consistent feedback (100 and 0% winning probability) were each presented 14 times; inconsistent symbols (66 and 33% winning probability) were each presented 27 times. Symbols were presented in a randomized order.

**TABLE 2:** Overview of experimental factors, number of symbols, procedure, and dependent measures in examining affective constructs and BOLD (Blood Oxygen Level Dependent) response for both experiments.

	Experiment 1	Experiment 2
Factors	Average feedback balance (positive/negative)	Average feedback balance (positive/negative)
	Feedback consistency (consistent/inconsistent)	Feedback consistency (consistent/ inconsistent)
	Time (Questionnaire Block 1/2/3)	
Number of symbols	Four (see Table 1 for more details)	Five (see Table 1 + control symbol)
Procedure	Practice Block (8 trials) Learning Block 1 (82 trials) PAQ Block 1 (4 trials) Learning Block 2 (82 trials) PAQ Block 2 (4 trials) Learning Block 3 (82 trials) PAQ Block 3 (4 trials)	Behavioral Practice Block (10 trials) Learning Block 1 (52 trials) Learning Block 2 (52 trials) Learning Block 3 (52 trials) fMRI Practice Block (5 trials) fMRI Block 1 (50 trials) fMRI Block 2 (50 trials) PAQ Block (4 trials)
Dependent measures	Current valence, current arousal, expected valence difference, expected arousal if gambling, expected arousal if passing	Current valence, current arousal, expected valence difference, expected arousal if gambling, expected arousal if passing; BOLD response

*PAQ, Predecisional Affect Questionnaire; fMRI, functional Magnet Resonance Imaging.*

##### Predecisional affect questionnaire block

Questionnaire blocks measured self-reported predecisional affect. In the questionnaires blocks, after looking at the symbol and before deciding for or against gambling, participants filled in a paper-pencil questionnaire (see Figure 2B for an example trial structure). In this questionnaire they rated their affective state from three different perspectives each on the dimensions of *valence* and *arousal* using a 9-point Self-Assessment Manikin Scale (SAM; Bradley and Lang, 1994). The first perspective asked participants to rate their current affective state: *“Please rate how you are feeling now.”* The second perspective asked them to rate their expected affective state if they would decide to gamble: *“Please rate how you would feel if you decided to gamble.”* The third perspective asked them to rate their expected affective state if they decide not to gamble: *“Please rate how you would feel if you decided not to gamble.”* Below each question we presented a SAM Valence Scale and a SAM Arousal Scale. Hence, we asked one question for each perspective but collected two ratings (valence, arousal) per perspective resulting in six variables: Current Valence/Arousal, Expected Valence/Arousal if gambling, and Expected Valence/Arousal if passing (see Table 2). In the questionnaire blocks each symbol was randomly presented once, resulting in four trials. For every symbol participants filled in the above mentioned questionnaire in a paper-pencil format. Taken together, we collected 24 ratings per questionnaire block. Question presentation was not randomized: they were first asked to rate their current affective state, then their expected affective state if gambling, and last their expected affective state if passing.

#### Procedure

At the beginning, participants were welcomed, filled in a demographic questionnaire, and gave their written informed consent to the experimental procedure. For an overview of experimental factors, dependent variables, and the chronological procedure (see Table 2). Participants could gain or lose points in the gambling task. They were informed that the 10 highest scoring participants would win 10 euros each. Each participant started the experiment with a balance of 500 points. The current score was presented after each block. Thus, participants got an immediate feedback after each block on how much points they won or lost in the preceding block. First, participants completed a practice block of eight trials which did not affect their balance. Then they started the first of the three learning blocks (for more details see section “Materials”). Participants indicated their gambling decision by pressing the assigned yes- or no-button. Key assignment was counterbalanced across participants. After each learning block there was a PAQ block (for more details see section “Materials”). Hence, we measured predecisional affect at three different time points which constituted the factor time (see Table 2). Participants could take a short self-timed break between blocks if they wanted to. At the very end, participants were debriefed.

### Results

First, we show that the experimental factors impact the self-report ratings. In a second step, we want to examine which self-reported affect variables predict gambling choices best. To begin with, we analyzed how the proposed valence constructs were correlated with one another. We used the rmcorr package in R (Bakdash and Marusich, 2017). Both expected valence variables (expected gambling and expected passing) were highly correlated (*r* = −0.53, *p* < 0.001), however, all other constructs were only moderately correlated (< *r* = 0.35). Therefore, we decided to compute a difference score for the expected valence perspectives (Expected Valence Difference = “Expected Valence if gambling”–“Expected Valence if passing”) as Charpentier et al. (2016) did to avoid collinearity in the analysis.

#### Expected Valence

We submitted the difference scores of self-reported expected valence ratings for the three TIMES and each symbol to a 3 × 2 × 2 repeated measures ANOVA. The factor BALANCE had two levels: positive, for symbols that won on average, and negative, for symbols that lost on average. The factor CONSISTENCY also had two levels, consistent, for symbols that returned consistent positive or negative feedback, and inconsistent, for symbols that returned mixed feedback. The three-way interaction TIME × BALANCE × CONSISTENCY was non-significant, *F*(2, 48) = 22.19, *p* = 0.07. However, the interaction BALANCE × CONSISTENCY turned out to be significant, *F*(1, 24) = 8.96, *p* = 0.006, η*_p_* = 0.272 (see Table 3). As it was a semi-disordinal interaction only the main effect BALANCE was interpretable, *F*(1, 24) = 22.19, *p* < 0.001, η*_p_* = 0.480. Symbols which had a positive balance, *M* = 1.273, *SE* = 0.307, had significantly higher difference scores than symbols which had a negative balance, *M* = −0.973, *SE* = 0.307, *p* < 0.001. Thus, the effect of a positive balance on expected valence ratings was even more pronounced for symbols returning consistent positive feedback in comparison to symbols returning inconsistent positive feedback.

**TABLE 3:** Estimated Marginal Means, Standard Errors (SE), and 95% Confidence Interval for the two-way interaction BALANCE × CONSISTENCY in the Analysis of Expected Valence Difference ratings.

				**95% Confidence Interval**	
Balance	Consistency	Mean	SE	Lower	Upper
Positive	Consistent	1.91	0.350	1.2082	2.605
	Inconsistent	0.64	0.350	−0.0585	1.338
Negative	Consistent	−1.08	0.350	−1.7785	−0.382
	Inconsistent	−0.87	0.350	−1.5651	−0.168

#### Current Valence

We submitted the self-reported current valence ratings to a 3 × 2 × 2 ANOVA for repeated measures. As in the expected valence analysis, we used the factors TIME, BALANCE, CONSISTENCY. The three-way interaction TIME × BALANCE × CONSISTENCY was significant, *F*(2, 48) = 6.49, *p* = 0.003, η*_p_* = 0.213 (for means and other statistics see Table 4). For resolving this interaction we conducted three additional 2 × 2 ANOVAs for repeated measures with the factors BALANCE and CONSISTENCY, one for each time point. For time 1, there were no significant differences between current valence ratings. However, for time 2, there was a significant CONSISTENCY × BALANCE interaction effect, *F*(1, 24) = 8.29, *p* = 0.008, η*_p_* = 0.257. *Post-hoc* Bonferroni corrected *t*-tests indicated that current valence ratings were smaller for consistent negative symbols compared to consistent positive symbols, *p* < 0.016. For time 3, there was a significant main effect for BALANCE, *F*(1, 24) = 11.45, *p* = 0.002, η*_p_* = 0.323. Symbols with an overall positive balance, *M* = 6.90, *SE* = 0.265, had higher current valence ratings than symbols with an overall negative balance, *M* = 6.28, *SE* = 0.265, irrespective of feedback consistency.

**TABLE 4:** Estimated Marginal Means, Standard Errors (SE), and 95% Confidence Interval for the threeway-way interaction TIME × BALANCE × CONSISTENCY in the Analysis of Current Valence ratings.

					95% Confidence Interval	
Time	Balance	Consistency	Mean	SE	Lower	Upper
Time 1	Positive	Consistent	6.24	0.285	5.67	6.81
		Inconsistent	6.60	0.285	6.03	7.17
	Negative	Consistent	6.52	0.285	5.95	7.09
		Inconsistent	6.40	0.285	5.83	6.97
Time 2	Positive	Consistent	6.92	0.285	6.35	7.49
		Inconsistent	6.24	0.285	5.67	6.81
	Negative	Consistent	6.08	0.285	5.51	6.65
		Inconsistent	6.56	0.285	5.99	7.13
Time 3	Positive	Consistent	7.12	0.285	6.55	7.69
		Inconsistent	6.68	0.285	6.11	7.25
	Negative	Consistent	6.28	0.285	5.71	6.85
		Inconsistent	6.28	0.285	5.71	6.85

#### Expected Arousal

Analogous to the expected valence analysis, we submitted the difference scores of self-reported expected arousal ratings for the three TIMES and each symbol varying in CONSISTENCY and BALANCE to a 3 × 2 × 2 repeated measures ANOVA. All interaction and main effects were non-significant.

#### Current Arousal

For self-reported current arousal we performed a 3 × 2 × 2 ANOVA for repeated measures with the factors TIME, BALANCE, and CONSISTENCY. All three- and two-way interactions were non-significant, however, the main effect of BALANCE was significant, *F*(1, 24) = 7.97, *p* = 0.009, η*_p_* = 0.249. Symbols with a positive balance, *M* = 4.39, *SE* = 0.29, had higher self-reported current arousal ratings than symbols with a negative balance, *M* = 3.97, *SE* = 0.29.

#### Choice Prediction

To test which affect variables predicted choice best, we ran a generalized mixed effects model using the glmer function from the lme4 package in R (Bates et al., 2015). As correlation analysis showed that both expected valence variables were highly correlated, we decided to compute a difference score to reduce collinearity. All other variables were only mildly correlated which is why we entered them separately into the model. We modeled the Participant ID as a random intercept and entered each affect variable as a fixed effect into the model resulting in the formula: Choice ∼ Difference Expected Valence + Current Valence + Expected Gambling Arousal + Expected Not Gambling Arousal + Current Arousal + (1| Participant ID). Significance was assessed via model comparison with an Alpha of 0.05. Expected Valence was the only significant predictor for gambling choice, β = 1.19, *SE* = 0.17, **X*^2^*(1) = 150.2, *p* < 0.001. This means, the higher the difference score of expected valence ratings (gambling—not gambling) were, the more likely participants chose to gamble. For more details regarding fixed and random effect structure (see Table 5).

**TABLE 5:** Generalized linear mixed effect estimates for the choice prediction model including the proposed affective predictors.

Predictors	Odds ratios	*CI*	*p*
(Intercept)	0.37	0.03–4.15	0.419
Difference expected valence	3.28	2.37–4.54	**<0.001**
(gambling–passing)			
Expected gambling arousal	1.05	0.76–1.45	0.760
Expected not gambling arousal	1.21	0.87–1.67	0.256
Current valence	1.24	0.92–1.68	0.156
Current arousal	0.80	0.55–1.16	0.233
**Random effects**			
σ^2^	3.29		
τ_00 Participant_	0.40		
ICC	0.11		
N_Participant_	25		
Observations	300		
Marginal *R*^2^/Conditional *R*^2^	0.733 / 0.762		

*Fixed Effects: Odds Ratios, Confidence Intervals (CI), and p-values. Random Effects: σ^2^ = within-person residual variance, τ_00 Participant_ = between-person variance, ICC = Proportion of variance explained by between-person differences; Marginal R^2^ = variance explained by fixed effects, Conditional R^2^ = variance explained by fixed and random effects. Significant results are printed in bold.*

### Discussion

In Experiment 1, we wanted to examine how subjective feelings are part of the decision process in a recurrent gambling task with unknown outcome probabilities. Hence, we developed a gambling task that was similar to the Iowa Gambling Task. However, our task varied feedback consistency, average feedback balance, and the learning experience in a systematic, controllable way. For measuring subjective feelings we took different classifications into account. Thus, we measured valence and arousal under the perspective of current and expected feelings. Our most important research question studied which of the proposed subjective feeling construct would predict choice. We found that expected valence was the only predictor for choices participants made. All other constructs were non-significant. Hence, the difference of expected valence ratings but not current valence or arousal constructs predicted choices which is in line with our hypotheses and previous research (Mellers et al., 1997; Charpentier et al., 2016; DeWall et al., 2016). At the same time, our findings challenge a decision guiding function of arousal. We found that self-reported current arousal indeed varied between good and bad symbols, however, it did not predict subsequent choices. As self-reported arousal ratings might be unreliable, it might be useful to simultaneously assess physiological arousal measures to enhance predictive power (Asutay et al., 2019). Future studies should further examine these findings and include physiological measures of autonomous activity instead of self-reported arousal in their choice prediction models.

Furthermore, we examined whether the measured variables were sufficiently different from one another. We found that most constructs correlated only mildly or moderately and, therefore, differed sufficiently. However, expected valence ratings of the two choice options were highly correlated which is why we computed a difference score for expected valence ratings (for a similar procedure, see Charpentier et al., 2016). Moreover, we examined how contextual factors like feedback consistency, learning experience, and average feedback balance influence the proposed subjective feelings variables. In general, we found that most self-reported ratings were influenced by contextual factors, yet, in different ways. Most constructs, except for current valence, were insensitive to time of measurement which implies a relatively early manifestation of constructs. As predicted, expected valence ratings distinguished between symbols with a positive and negative balance and for positive balanced symbols also between consistent and inconsistent symbols. This was the case over all measurement points, indicating an early manifestation of expectancy constructs. The impact of contextual factors on current valence ratings changed over time. After the first block there was no significant difference between ratings, at time 2 consistent positive symbols were rated higher than all other symbols, at time 3 positive balanced symbols (consistent and inconsistent) had higher current valence ratings than negative balanced symbols. In other words, current valence changed as participants learned symbol-feedback contingencies. This posits that current valence manifests over time as learning takes place. The difference of expected arousal ratings was not affected by contextual factors. Taken together, contextual factors influenced most of the proposed constructs but not expected arousal ratings.

## Experiment 2

In Experiment 1 we found that self-reported affect ratings were influenced in different ways by feedback consistency, feedback balance, and learning experience. As a next step, we wanted to examine how these contextual factors determine predecisional affective brain activity. Lindquist et al. (2016) tested three competing hypotheses regarding neural representation of affect in a meta-analysis of the human neuroimaging literature. The bipolarity hypothesis assumes that pleasant and unpleasant feelings are endorsed by a brain system that monotonically increases and decreases along the valence dimension. Second, the bivalent hypothesis posits two independent brain systems for positive and negative affect. Last, the affective workspace hypothesis suggests that valence is best represented on a neuronal level as a valence general neural workspace which recruits a flexible set of valence-general areas. Results clearly favored the affective workspace hypothesis while evidence for both other theories was rather weak. Valence-general activations were found in the bilateral anterior insula, thalamus, dorsal ACC, bilateral lateral orbitofrontal cortex, supplementary motor area, bilateral amygdala, the ventral striatum, dorsomedial prefrontal cortex, bilateral ventro-lateral prefrontal cortex, and lateral portions of the right temporal/occipital cortex. At the same time, the authors acknowledge that it might be a possibility that the arousal component of affect might contribute to valence-general activation patterns as separating arousal from valence is both a statistically and theoretically complex endeavor.

As in Experiment 1, our task design varied the symbol’s balance and its feedback consistency. In accordance with the hypothesis of a valence-general affective workspace, we expected that all symbols, which varied in contextual factors and therefore also in affect ratings, recruit the same brain regions. In line with the presented evidence we supposed to find brain activity in the anterior cingulate cortex, the accumbens area, the thalamus, the amygdala, the insula, and the prefrontal cortex. In addition to that, we hypothesized that the symbol’s balance or its feedback consistency would have a rather small or no effect on observed brain activity as valence-general brain regions work together to produce different valence intensities.

### Materials and Methods

#### Participants

Data were collected from 22 adults of which five (*M*_age_ = 50.4 years, *SD* = 2.7 years, two men) were excluded because they had not learned the symbol-feedback contingencies after the third block. Exclusion criteria were set at a gambling rate below 70% for the 100% chance condition as well as a gambling rate above 30% for the 0% chance condition. The final sample consisted of 17 adults (six men) aged between 20 and 57 (*M*_age_ = 35.5 years, *SD* = 12.0 years). Hence, the dropout seems to be age-related, meaning that older participants had difficulties learning the symbol-feedback contingencies. Furthermore, after the MRI block one participant decided to end the study. Therefore, the sample for the questionnaire block comprised 16 adults (six men) aged between 20 and 57 (*M*_age_ = 36.4 years, *SD* = 11.8 years). Participants had normal or corrected to normal vision; 16 participants were right-handed, one was left-handed; six participants had at least an educational degree of a German high school diploma, whereas the others had a German Middle School Degree.

The study was conducted in accordance with the Declaration of Helsinki. Participants gave their written informed consent and were told that they could refrain from the study at any point without consequences. The study protocol was approved by the ethics committee of the Hannover Medical School with the study ID 7416.

#### Materials

The general materials did not change much in comparision to Experiment 1, however, we adapted the procedure and timing parameters to the needs of the fMRI setting. In all blocks (learning, fMRI, and PAQ Block) participants did not respond to the symbol directly but rather to the question *“Do you want to gamble?”* as presented in Figure 2C. We thought that it would be easier for participants to have a consistent task structure. Moreover, we introduced a control symbol to the gambling task, which regardless of choice did not affect participants’ score. As we wanted to isolate affect-related brain activity, we needed the control symbol to compute difference contrasts (see “Data Analysis” section for more details). Finally, we decided to use real money instead of points because we hoped this would result in stronger neural activations. Participants could win or lose 20 cents in each trial. “Gamble” and “Pass” key assignment was counterbalanced over participants. For stimulus presentation, we used the software NBS Presentation^2^.

The learning blocks consisted of 52 randomized trials: eight consistent-positive feedback symbols, eight consistent-negative feedback symbols, eight control symbols, 14 inconsistent-positive feedback symbols and 14 inconsistent-negative feedback symbols. We decided to present more inconsistent symbols to make it easier for participants to learn these contingencies. Each trial had the following timing parameters: fixation asterisk (250 ms), symbol (500 ms), fixation asterisk (500–800 ms), choice (“*Do you want to gamble?*,” until response), feedback depending on the decision and the symbol’s winning probability (750 ms), inter trial interval (ITI; 500–800 ms).

**FIGURE 3:**
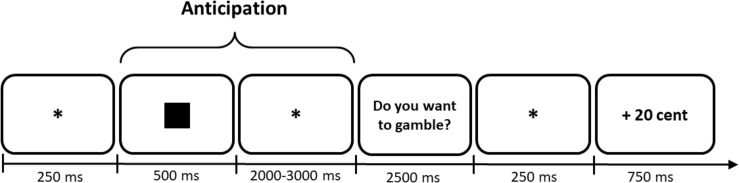
Example trial to illustrate the timing of the fMRI Monetary Gambling Task. Numbers characterize presentation durations in ms. In this case the participant would have chosen to gamble and subsequently won 20 cents. Only the anticipation period was used for analysis of brain activity. *Indicates a fixation dot.

The fMRI blocks consisted of 50 randomized trials (10 repetitions of each symbol). We also adjusted the timing parameters to fit the fMRI method (see Figure 3). The inter trial interval (ITI) and the anticipation period were both jittered with an average duration of 2,500 ms, ranging from 2,000 to 3,000 ms. Participants could answer within 2,500 ms when they were requested to indicate their decision (see Figure 3). If they did not respond within this time frame, *“XXX”* appeared as feedback which did not affect their momentary balance. However, participants were still asked to give an answer although a no-decision would have yielded a similar result for the participant’s overall balance. We did so to reduce missed trials, which cannot be used in analysis, to a minimum.

Last, in the PAQ Block we did not change trial structure (see Figure 2B). However, we presented five symbols instead of four, as we had one additional control symbol in Experiment 2. Again, participants indicated their predecisional affective states on a paper-pencil questionnaire. Hence, the PAQ Block consisted of five trials (see Table 2).

#### Procedure

For an overview of experimental factors and the procedure in comparison to Experiment 1 (see Table 2). First, participants were welcomed, filled in a demographic questionnaire, and gave their written informed consent to the experimental procedure. We placed five euros in front of each participant to underline that they could win and lose real money during the experiment. Each participant started the experiment with a balance of five euros. The participant’s current money balance was presented after each block. Thus, participants got an immediate feedback after each block how much they won or lost in the respective block. Practice blocks did not affect the participants’ balance in any part of the experiment. After completing the practice block consisting of 10 trials (each symbol was presented twice), they started the three learning blocks. Participants could indicate their gambling decision by pressing the assigned gamble or pass button on a two keyed Cedrus Response Box (RB-380). In the learning phase, participants could take a short self-timed break between blocks if they wanted to. Before each task change, participants completed a practice block to get used to the procedure or the changed trial timing. After the fMRI practice block (five trials), they completed two fMRI blocks starting with a 6 s fixation trial. Between both blocks a 50 s break was inserted and the balance of monetary gains or losses in the preceding block was presented. In this phase we used NordicNeuroLab’s VisualSystem for stimulus presentation in the MRI scanner and ResponseGrip to collect their answers. The VisualSystem goggles were placed on the head coil where participants could adjust the visual acuity depending on their visual condition. Participants were instructed to use their right and left thumb to indicate their decision. After the fMRI phase, participants completed the PAQ Block. At the very end, participants were debriefed and got paid their overall balance.

#### fMRI Data Acquisition

Data were collected using a 1.5 T Magnetom Avanto scanner (Siemens Medical Systems, Erlangen, Germany) with an 18-channel head coil. Functional images were obtained using a T2^∗^-weighted echo planar imaging (EPI) sequence with TR = 2,000 ms, TE = 35 ms and flip angle = 80°, 498 volumes, resulting in a duration of 16.6 min. Each functional image consisted of 23 axial slices, with 64 × 64 matrix, 200 mm × 200 mm field of view (FOV), 5 mm thickness, 1 mm gap, and 3.125 mm × 3.125 mm inplane resolution. Structural images were obtained using a 3D structural sagittal T1-weighted MPRAGE image. Each structural image consisted of 192 contiguous slices, with 256 mm × 250 mm matrix size and 1 mm slice thickness.

#### Data Analysis

##### Behavioral

Behavioral data were analyzed by computing a repeated measures ANOVA for each dependent variable of interest as we did in the behavioral analysis of Experiment 1. As we had a small sample size and just one measurement for each affect construct, we decided to skip the choice prediction analysis.

##### fMRI

Data were preprocessed and analyzed using SPM12^3^. The first three volumes were discarded due to longitudinal magnetization equilibration effects. First, structural and functional images were roughly reoriented using the EPI-derived MNI template (ICBM 305, Montreal Neurological Institute). After realignment, the structural images were coregistered to the EPI images, and the six movement parameters (x, y, z, pitch, yaw, roll) saved to include them as covariates in the first level analysis. Then EPI images were time shifted to the middle slice to correct differences in slice acquisition timing. In a further step, both structural and functional images were directly normalized to the MNI template. The normalized EPI images were smoothed with a Gaussian kernel of 8 mm full-width half-maximum (FWHM) and filtered with a high-pass filter of 128 s.

In the first level analysis, we specified conditions, estimated parameters, and computed contrasts for each participant using the canonical hemodynamic response function (HRF) and a general linear model. Therefore, we defined the time-locked anticipation periods (see Figure 3) of the five symbols as regressors and included the six motion parameters as covariates to reduce signal-corrected motion effects. Regardless of the later gambling decision, anticipation periods of the symbols were each modeled as a separate regressor. Response, feedback, and between-block pause periods were still modeled but not included in the analysis. Additionally, anticipation periods of missed trials were treated the same way, the ITI serving as implicit baseline. Then, we applied classical parameter estimation with a one-lag autoregressive model and a masking threshold of 0.8 to minimize false positive voxels. Finally, we computed the t-contrasts of the symbols compared to the control symbol to isolate brain activity of potential wins and losses in the anticipation period. Thus, we computed the contrasts “positive-consistent > control,” “positive-inconsistent > control,” “negative-inconsistent > control,” and “negative-consistent > control” to take them to the second level group analysis.

In the second level group analysis, we defined a 2 × 2 full factorial design for repeated measures with the factors BALANCE and CONSISTENCY while AGE was included as a covariate due to the previously discovered age related dropouts caused by learning difficulties. We assigned the factor levels in the same way as in the behavioral analysis. For computation, we entered each participant’s t-contrasts of each symbol in comparison to the control condition which were calculated in the first level analysis. For each factor, variances were assumed to be unequal and independence was not given. Furthermore, we applied implicit masking and carried out a classical parameter estimation.

### Results

#### Behavioral

##### Expected valence

We submitted the difference scores of self-reported expected valence ratings for each symbol to a 2 × 2 repeated measures ANOVA as in Experiment 1. The interaction BALANCE × CONSISTENCY turned out to be significant, *F*(1, 15) = 4.98, *p* = 0.041, η*_p_* = 0.241. As it was a semi-disordinal interaction, only the main effect BALANCE was interpretable, *F*(1, 15) = 13.63, *p* = 0.002, η*_p_* = 0.476. Symbols which had a positive balance, *M* = 2.06, *SE* = 0.558, had significantly higher difference scores than symbols which had a negative balance, *M* = −1.16, *SE* = 0.558. Thus, the effect of a positive balance on expected valence ratings was even more pronounced for symbols returning consistent positive feedback in comparison to symbols returning inconsistent positive feedback.

##### Current valence

We submitted the self-reported current valence ratings to a 2 × 2 ANOVA for repeated measures. The main effect CONSISTENCY proved to be significant, *F*(1, 15) = 6.05, *p* = 0.027, η*_p_* = 0.287, with consistent symbols, *M* = 7.44, *SE* = 0.334, having significantly higher current valence ratings than inconsistent symbols, *M* = 6.72, *SE* = 0.334. The main effect BALANCE was only marginally significant, *F*(1, 15) = 4.45, *p* = 0.052, η*_p_* = 0.229.

##### Expected arousal

As before, we submitted the difference scores of self-reported expected arousal ratings for each symbol varying in CONSISTENCY and BALANCE to a 2 × 2 repeated measures ANOVA. All interaction and main effects were non-significant.

##### Current arousal

For self-reported current arousal we performed a 2 × 2 ANOVA for repeated measures with the factors BALANCE and CONSISTENCY. The two-way interaction was non-significant, *F*(1, 15) < 1, however, the main effect BALANCE reached significance, *F*(1, 15) = 11.50, *p* = 0.004, η*_p_* = 0.434. Symbols with a positive balance, *M* = 4.16, *SE* = 0.447, had significantly higher self-reported current arousal ratings than symbols with a negative balance, *M* = 3.31, *SE* = 0.447.

#### fMRI

**FIGURE 4:**
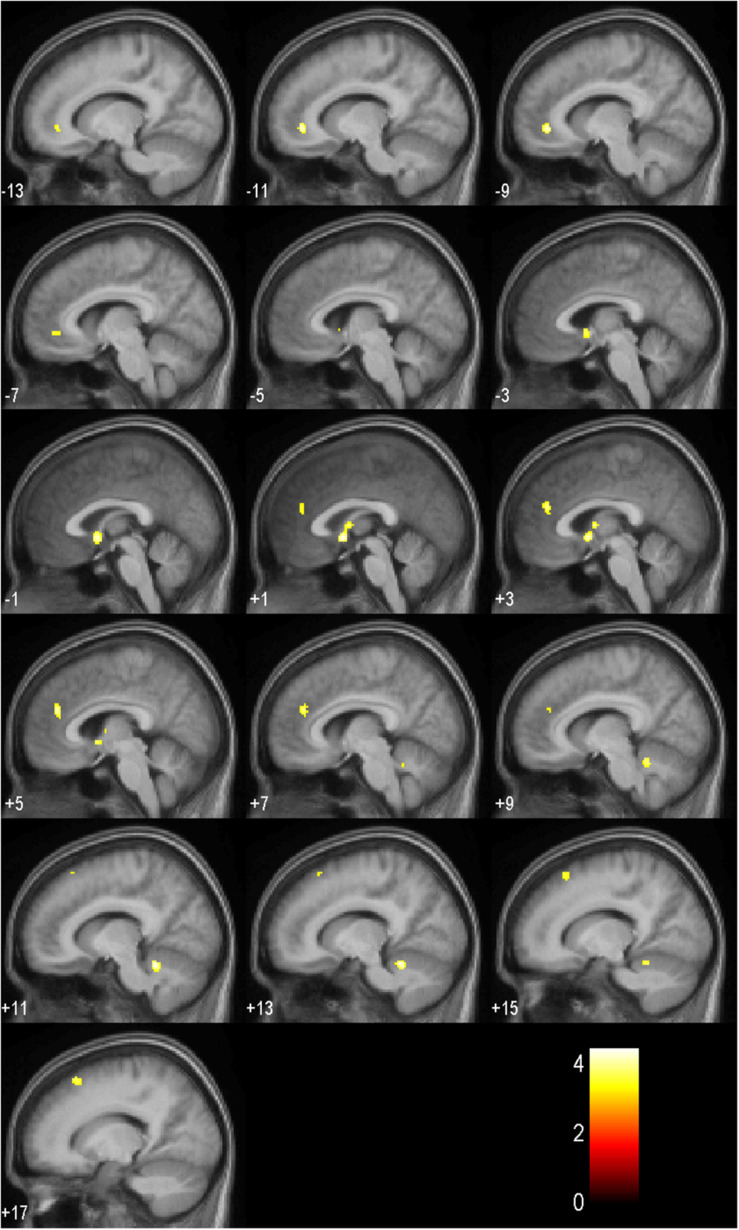
Activation patterns in the anticipation period as listed in Table 6 for all symbols in comparison to the control symbol (*p* < 0.001, uncorrected; *n* = 17). Positive values represent the number of sagittal slices from the center to the right hemisphere. Negative values indicate the number of sagittal slices from the center to the left hemisphere. The colored bar specifies the respective *t*-value’s magnitude.

**FIGURE 5:**
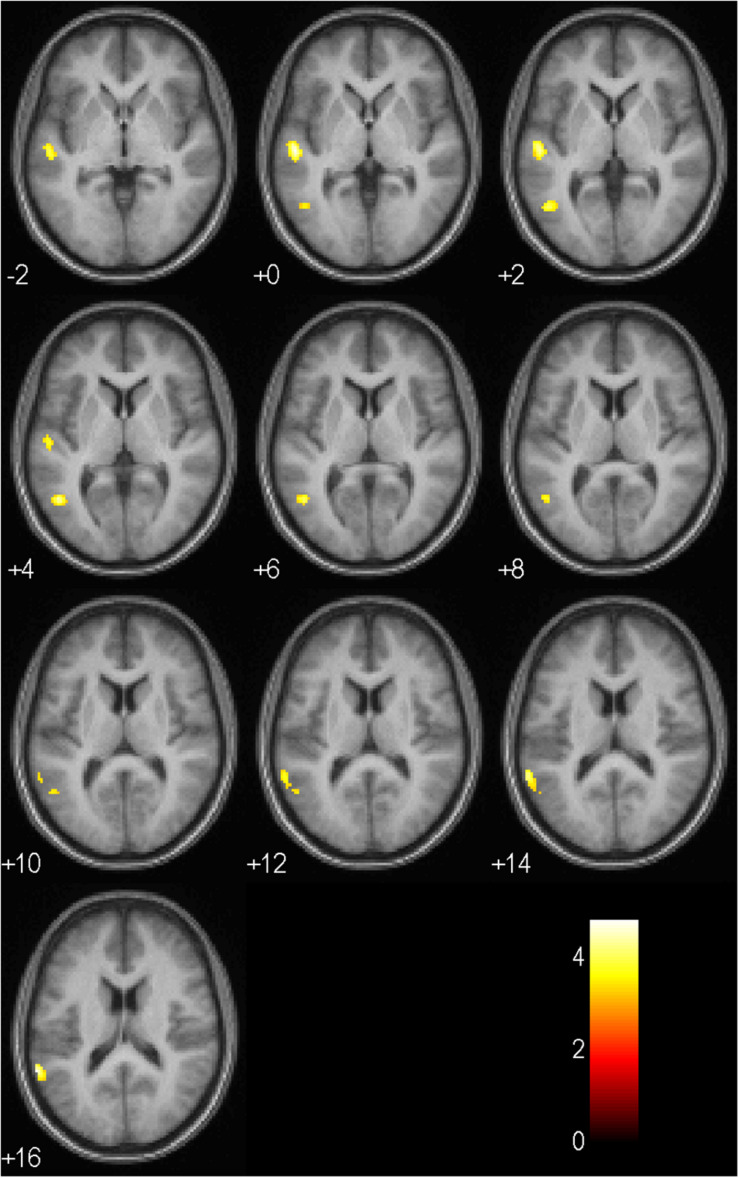
Activation patterns in the anticipation period as listed in Table 6 for negative balanced in comparison to positive balanced symbols (main effect balance, no > yes; *p* < 0.001, uncorrected; *n* = 17). Positive values represent the number of axial slices from the center downwards. Negative values indicate the number of sagittal slices from the center upwards. The colored bar specifies the respective *t*-value’s magnitude.

Results of the full factorial analysis are presented in Table 6 and Figures 4, 5 at *p* < 0.001 (uncorrected) and a minimum voxel cluster of 40. Despite the possibility of false positive results, we decided to conduct the analysis to give an idea of potentially activated brain regions. Main and interaction effects which are not reported did not approach significance. For all symbols, which returned positive or negative feedback in comparison to a control symbol, which regardless of choice returned a null feedback, we found general activations in the anticipation period. As presented in Figure 4, symbols associated with positive or negative feedback showed more activity in the anticipation period in the Cerebellum Exterior, the Accumbens Area, the Thalamus Proper, the Anterior Cingulate Cortex, the Medial Superior Frontal Cortex, and the Superior Frontal Cortex. Furthermore, we found that negative balanced symbols produced stronger activations in the Superior Temporal and Middle Temporal Cortex in the anticipation period in comparison to positive balanced symbols (see Figure 5).

**TABLE 6:** Group maximum *T*-values and MNI Coordinates of activation foci for the t-contrast Condition (general activation averaged over anticipation periods of the four symbols; *p* < 0.001, uncorrected; *n* = 17) and the t-contrast Balance (negative > positive; *p* < 0.001, uncorrected; *n* = 17).

Region	H	*x*	*y*	*Z*	*t*	Size
**Condition**						
Cerebellum exterior	R	12	–46	−20	4.45	49
Accumbens area	L	0	4	−4	4.40	49
Thalamus proper	R	2	−2	6	4.05	44
Anterior cingulate	L	–10	40	−4	4.25	43
Medial superior frontal	R	4	40	24	4.07	60
Superior frontal	R	18	20	56	3.73	56
**Balance**						
Superior temporal	L	–54	–20	0	4.80	91
		–64	–48	16	4.79	52
Middle temporal	L	–48	–60	4	4.47	65
		–52	–60	12	3.53	45

*Size, number of activated voxels; L, left; R, right; H, hemisphere.*

### Discussion

In Experiment 2 we adapted the gambling task from Experiment 1 to the fMRI environment. We were interested in predecisional affect in the anticipation period of a complex decision-making task. Therefore, we wanted to exploratively examine affective brain-activity and varied feedback consistency and average feedback balance. We could only show brain activity for uncorrected *p*-values which is due to our small sample size. Nevertheless, we think that our exploratory results are still worth reporting since future hypothesis testing research can use our findings as a starting point. In line with previous research (Lindquist et al., 2016), we could observe most activity in the valence-general condition which indicated activity independent of experimental factors in the accumbens area, thalamus proper, anterior cingulate cortex, medial superior frontal cortex, superior frontal cortex, and the cerebellum exterior. A negative average feedback balance produced activity in the superior temporal and middle temporal gyrus. Analysis of expected valence ratings replicated findings from Experiment 1 meaning that expected valence ratings differed between positive and negative balanced symbols and for positive balanced symbols between consistent and inconsistent symbols. Findings of current arousal could also be replicated with positively balanced symbols having higher arousal ratings than negatively balanced symbols. Findings of expected arousal and current valence could only partially replicate findings from Experiment 1. Overall, the presented evidence suggests that valence-general regions are also recruited in the anticipation period of our decision-making task. During the anticipation period self-reported expected valence and current arousal ratings are robustly influenced by contextual factors.

Our results indicate some overlapping activity with results of the meta-analysis by Lindquist et al. (2016). However, there is still a considerable difference between valence-general active regions as we did not find activity in the amygdala, the insula, and prefrontal regions, for example. The absence of activity in prefrontal regions could be explained by findings from Oldham et al. (2018). They conducted a meta-analysis on fMRI studies that used the monetary incentive delay task. This task makes it possible to disentangle the anticipation period from the feedback period as well as gains from losses. Their findings suggest that there is great overlapping neural activity between the anticipation of gains and losses including the amygdala, thalamus, striatum, and insula which is in line with Lindquist et al.’s (2016) findings and the affective work-space hypothesis. Furthermore, activity in orbitofrontal/ventromedial prefrontal regions was only observed during the reward feedback period which could explain the absence of activity in our findings. In general, Wilson et al. (2018) replicated the findings in another meta-analysis and analyzed active brain regions in more detail. This resulted in similar activations like we found adding activity in the cerebellum, the superior frontal gyrus, and the medial superior frontal gyrus to the meta-analytic evidence. However, we could not observe activity in the insula and the amygdala which has been a robust finding in the presented meta-analyses (Lindquist et al., 2016; Oldham et al., 2018; Wilson et al., 2018). Both brain areas have been identified as key nodes of the so called salience network which appears to serve the function of detecting novel stimuli across different modalities (Uddin, 2015). As we examined neural activity in relation to a control symbol, this could be the reason why we did not observe neutral activity in these areas of the salience network. Participants continuously viewed different symbols intermitted by fixation asterisks and the feedback presentation. We argue that the recognition of the control symbol, like all other symbols, also elicited a salience response. Hence, the control symbol was as novel as the other symbols in our experimental design which resulted in no greater or lesser neural activity in the salience regions.

## General Discussion

In two experiments, we examined the involvement of subjective feelings in the decision-making process. We studied how contextual factors influence current and expected subjective feelings and which constructs predict choice behavior best. We addressed the problems of common research designs like the IGT (Dunn et al., 2006) by developing a recurrent decision task that can vary contextual factors (feedback consistency, average feedback balance, learning experience) in a systematic way. Furthermore, we presented only one symbol at a time and could, therefore, solve the previously mentioned problem of the IGT without losing ecological validity. To our knowledge this is the first study that took a two-dimensional affect approach (valence, arousal) for measuring self-reported expected and current affect in a recurrent decision task. This provides a fuller picture of involved affective processes in recurrent decision-making. Furthermore, we exploratively looked at neural activations depending on contextual factors. Our results suggest that expected valence is the main and only self-reported subjective feeling component that predicts decisions. Hence, self-reported expected valence yet not self-reported current affect predicted decisions. Additionally, we observed valence-general neural activity in Experiment 2 while participants’ self-reported expected valence depended on contextual factors. Although self-reported current affect ratings also depended on contextual factors, the observed effect size and effect consistency for expected valence was substantially bigger. In sum, we observed valence-general activity in line with the presented meta analyses (Lindquist et al., 2016; Oldham et al., 2018; Wilson et al., 2018) and observed inconsistent and smaller contextual effects for self-reported current valence than for self-reported expected valence.

We carefully interpret our findings in the way that based on past experiences symbols induced current affect (fMRI findings, self-report current affect ratings) which in turn prompted further cognitive processes like expectancies of future outcomes (expected valence). If this is the case, participants would feel something and use this feeling to build their expectancies upon this feeling which is reflected in differential expected affect ratings. However, we did not find clear self-reported current affect patterns and no high correlation between current valence and expected valence which limits our interpretation. The reason for this contradiction could be the way we asked for current affect. Västfjäll and Slovic (2013) suggest, additionally to the proposed dimensions, to distinguish *incidential affect*, which is unrelated to the decision problem, from *integral affect* which is inherently linked to the decision problem. Hence, to get a more sensitive measure of incidential current affect we might have asked participants how they felt while seeing the symbol. This might have led to more consistent findings and a bigger predictive power of current affect. Keeping this in mind, we should be careful with this interpretation as our findings have limitations that make it impossible to draw final conclusions. Future research should focus on how current and expected affect interact or do not interact with each other. Experimental designs would have to make sure that the measurement of current affect is more precise and should examine whether it is even possible to separately manipulate expected affect and current affect. If it is not possible, this will provide more evidence for the described interpretation.

Complementing our interpretation, Moors and Fischer (2019) suggest that from a theoretical perspective there is no need to assume an intervening emotion variable to cause behavior. Even cases incorporating maladaptive emotions can be reinterpreted in a goal-directed way. For example, a student is paralyzed during her presentation in class as she is afraid. Her goal could be not to make a mistake which she tries to control through increased self-monitoring (Clark and Wells, 1995). This limits her cognitive resources which makes committing mistakes more likely. Thus, she tries to control the first mistake with increased self-monitoring. This results in a vicious cycle which eventually paralyzes her. In her logic, if she stops speaking, she cannot make mistakes which is her overarching goal. Consequently, in this model of explaining the student’s behavior, there is no need of a mediating emotion variable. The authors conclude that “emotions may point in imprecise ways to other factors (values and expectancies) that do the actual causal work. If so, it may be time to replace explanations in terms of emotions with explanations in terms of these other factors” (Moors and Fischer, 2019, p. 98). In our findings we can also see that expectancies are a much better predictor than current affect. Incorporating subjective feelings and expectancies of subjective feelings into one model shows that there is no predictive power of current affect as a goal-directed account of emotions would predict.

As mentioned before, there are some limitations to our study we would like to address now. First, in the fMRI analysis we report uncorrected *p*-values. Hence, cumulated alpha errors could have led to false positive results. However, we conducted an explorative fMRI analysis which is useful for generating hypotheses and should not be taken as conclusive knowledge. Adding to that, we would like to point out that we had a relatively small sample size in both experiments which underlines the robustness of effects regarding expected valence. However, it is still possible that we missed smaller effects due to the low power of our study. Future research should replicate the main findings with a larger sample size. Moreover, we did not present questions in a randomized order which could have led to systematic biases in affect ratings. Future research should counterbalance or randomize question presentation to make sure that there is no hidden bias. We expect though that findings regarding affect ratings will not change in a meaningful way. Taken together, our findings can only be preliminary due to the described limitations. Nevertheless, we still think that our results make a valuable contribution to inspire future research and neurocognitive decision theories.

Future research should also measure current affect in a more sensitive way as we proposed before. This would be the first step to further study how current and expected affect might work together. Furthermore, experimental designs should try to separately manipulate current affect and expected affect. We have two ideas how this could be accomplished. First, we could present symbol-feedback contingencies in the beginning and start with a questionnaire block. This would mean that participants have not experienced any outcome but draw on their knowledge and should therefore report differential valence expectancy ratings. Following reinforcement learning models (Holroyd and Coles, 2002), having not experienced an outcome before, might eliminate current affective experiences when viewing the symbol. A second option would be to switch symbol-feedback contingencies after a learning phase and before a questionnaire phase. Participants should be told which symbols have changed, so that they could adjust their expectations accordingly. This way participants would have current affect ratings based on their learning history and expected valence ratings based on the new information they received. Studying how these changes in experimental design affect subsequent gambling decisions could elucidate how current and expected affect work together and which is causal for decisions. Moreover, we would like to point out that self-reported arousal might not be the best way to measure an emotional arousal component as it produces inconsistent results (Asutay et al., 2019). It might be better to additionally use physiological arousal measures.

## Conclusion

Examining the relations among current and expected affective constructs in causing decision is a sensible way for future theorizing and empirical research on the affective involvement in decision-making.

## Data Availability Statement

The raw data supporting the conclusions of this article will be made available by the authors, without undue reservation, to any qualified researcher.

## Ethics Statement

The studies involving human participants were reviewed and approved by the ethics committee of the Hannover Medical School. The patients/participants provided their written informed consent to participate in this study.

## Author Contributions

DJ, JR, and MB designed the study. DJ and MB collected and analyzed the data, DJ, MB, JR, and JDR wrote the manuscript. All authors approved the final version of the manuscript.

## Conflict of Interest

The authors declare that the research was conducted in the absence of any commercial or financial relationships that could be construed as a potential conflict of interest.

## Footnotes

1 http://www.neurobs.com

2 http://www.neurobs.com

3 http://www.fil.ion.ucl.ac.uk/spm/
